# Surgical Management of Extraskeletal Chondrosarcoma in the Trapezius Muscle: A Report of a Rare Case

**DOI:** 10.7759/cureus.74318

**Published:** 2024-11-23

**Authors:** Arın Celayir, Baran Sevgil, Mahmut K Ozsahin, Huseyin Botanlioglu

**Affiliations:** 1 Orthopaedics and Traumatology, Istanbul University-Cerrahpasa, Cerrahpasa Medical Faculty, Istanbul, TUR

**Keywords:** chemotherapy, extraskeletal chondrosarcoma, magnetic resonance imaging, radiotherapy, wide resection

## Abstract

Extraskeletal chondrosarcomas are malignant tumors that develop in soft tissues rather than in bones. Unlike skeletal chondrosarcomas, which are more common, these tumors can occur in areas such as muscles, tendons, or fat. Their unique location in the body makes them distinct and sometimes challenging to diagnose and manage effectively. In this article, we will describe the surgical treatment of a patient diagnosed with extraskeletal chondrosarcoma within the trapezius muscle.

## Introduction

Extraskeletal chondrosarcomas are rare, malignant tumors that originate in soft tissues rather than in bones, distinguishing them from the more common skeletal chondrosarcomas. These tumors can develop in various soft tissue locations such as muscles, tendons, or fat, making them a unique and challenging subset of chondrosarcomas [1]. They are characterized by the production of cartilage-like tissue, and although they are rare, they tend to be aggressive and require prompt and comprehensive treatment. Diagnosing extraskeletal chondrosarcomas can be challenging due to their potential to mimic other soft tissue sarcomas, necessitating thorough imaging studies and histopathological evaluation for accurate identification.

The main treatment for extraskeletal chondrosarcomas is surgical excision with the goal of removing the tumor entirely and achieving clear margins to reduce the risk of local recurrence. When complete resection is not possible or the tumor is in a critical location, additional treatments may be needed. Radiation therapy can help manage local disease and lower recurrence risk, especially in cases with positive surgical margins. Chemotherapy, although not always effective, may be considered for high-grade tumors or those that have spread [2].

The management of extraskeletal chondrosarcomas often involves a multidisciplinary approach, integrating surgical, medical, and radiation oncology expertise to optimize outcomes. Despite aggressive treatment, these tumors have a propensity for local recurrence and distant metastasis, underscoring the importance of long-term follow-up and surveillance [3]. Understanding the behavior, diagnostic challenges, and treatment strategies for extraskeletal chondrosarcomas is crucial for improving patient outcomes and advancing the field of sarcoma research.

## Case presentation

**Figure 1:**
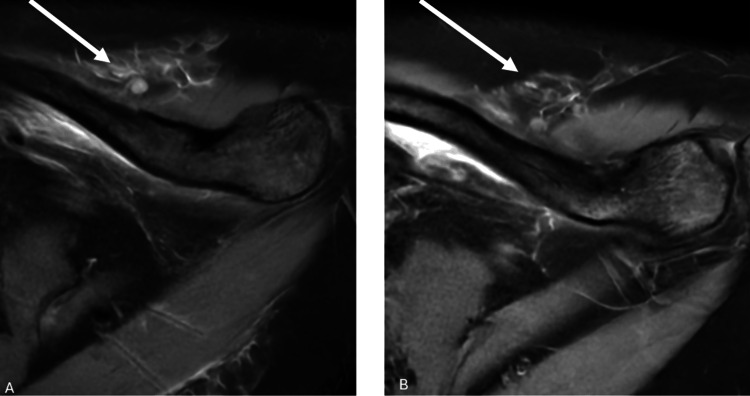
(A-B) Axial magnetic resonance images of the patient. The white arrow shows the lesion in the relevant magnetic resonance images.

**Figure 2:**
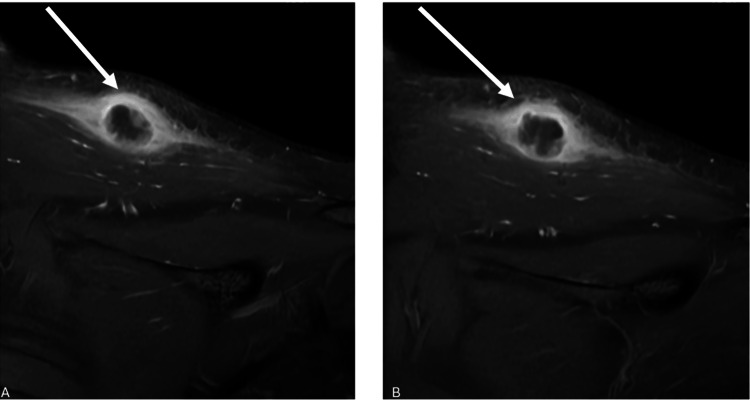
(A-B) Coronal magnetic resonance images of the patient. The white arrow shows the lesion in the relevant magnetic resonance images.

A 26-year-old female patient presented to our clinic with complaints of pain in the left shoulder. The patient has no known additional chronic illnesses. Informed consent was obtained before performing any procedures. During the examination, a 1x2 cm palpable mass was found in the left scapular region. In the patient's medical history, there was a biopsy performed on the left scapular region at an external center three months ago. After requesting current radiographs, a contrast-enhanced MRI of the left shoulder was ordered. The MRI scans revealed an 18x18 mm mass within the left trapezius muscle (Figures 1-2).

**Figure 3:**
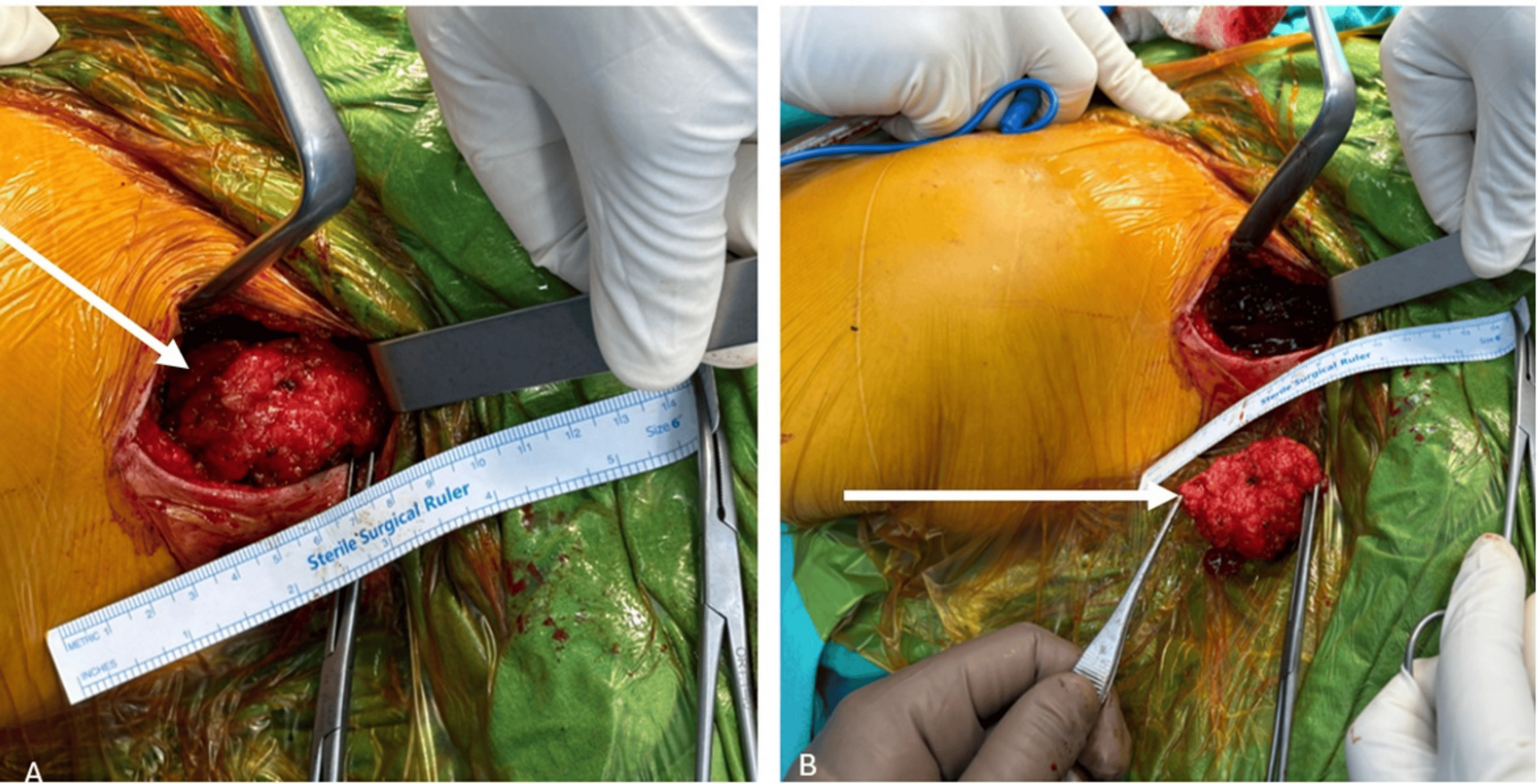
Perioperative images of the lesion. (A) Lesion before removal; (B) Lesion after removal. The white arrow indicates the lesion during the surgical procedure, while it is inside the trapezius muscle, and after it has been removed.

**Figure 4:**
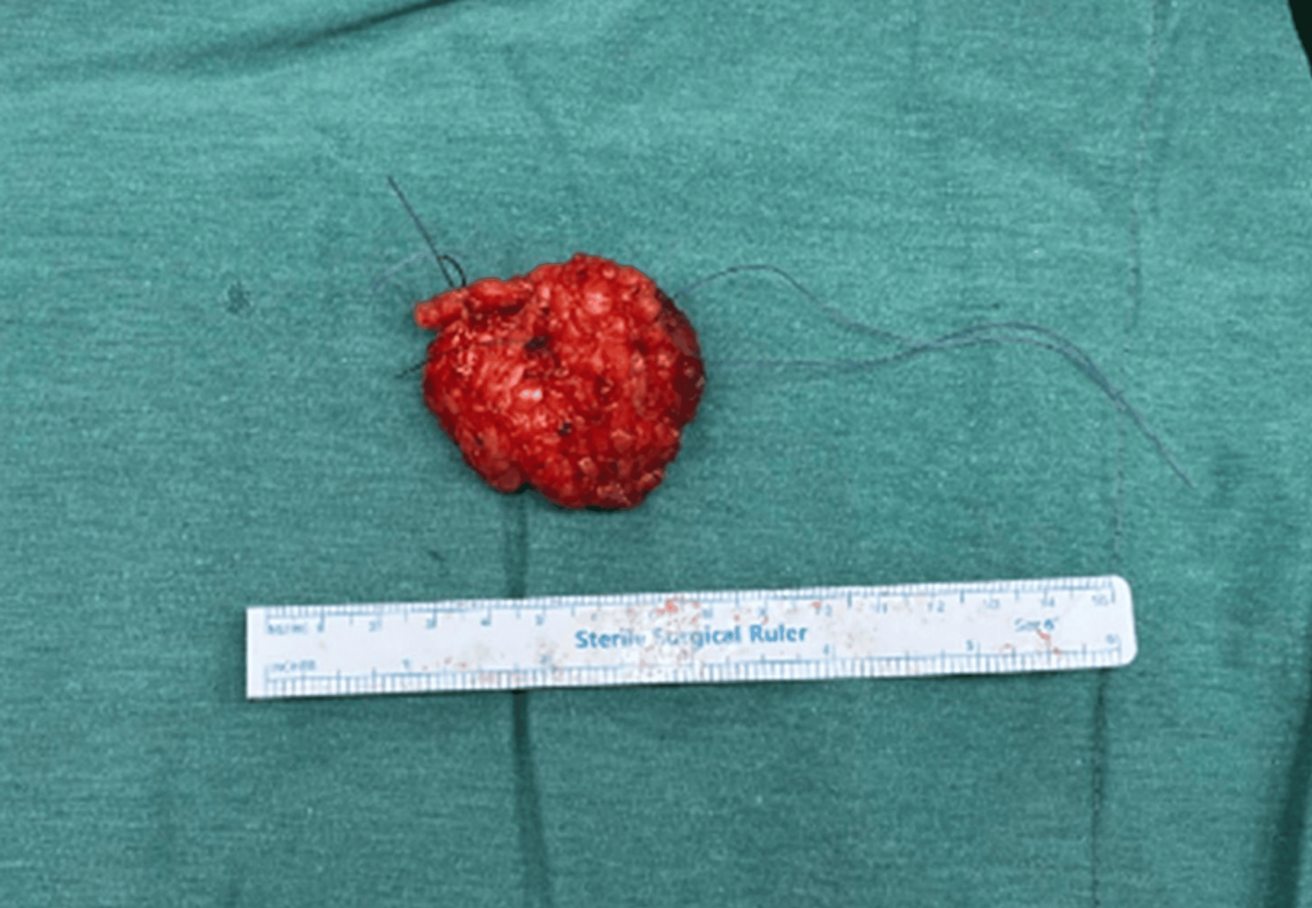
Macroscopic image of the lesion after the wide resection.

The biopsy sample taken at the external center was brought to our faculty, and our pathologists requested a slide consultation. The result was reported as extraskeletal chondrosarcoma. No metastasis was detected in the positron emission tomography (PET) scan performed at the external center. A wide resection surgery was planned for the patient. The patient was operated on in a beach chair position. A posterior incision was made in the left shoulder. The trapezius muscle was reached. The mass was identified, and five samples from the margins were sent for frozen section analysis. Since the frozen section margins were negative, the excised mass was sent to pathology (Figures 3-4).

The surgery was concluded, and a shoulder arm sling was applied to the patient. The patient was discharged the next day and called for a follow-up. The pathology result confirmed extraskeletal chondrosarcoma. The patient was consulted with medical and radiation oncology, and no chemotherapy or radiotherapy was planned.

## Discussion

Extraskeletal chondrosarcoma is a rare and malignant tumor that originates in soft tissues, and its genetic underpinnings are still being explored. While the precise genetic mechanisms driving these tumors are not fully understood, research has identified several genetic abnormalities associated with chondrosarcomas in general [4]. Mutations in genes such as IDH1 and IDH2, which are involved in cellular metabolism, have been frequently observed in both skeletal and extraskeletal chondrosarcomas. Additionally, alterations in the EXT1 and EXT2 genes, which play a role in heparan sulfate biosynthesis, have been linked to the development of these tumors. Chromosomal abnormalities, such as ring chromosomes and amplifications of certain genomic regions, have also been reported. Our patient also had mutations in the IDH1 and IDH2 genes. It is expected that these mutations are present in most cases of extraskeletal chondrosarcoma.

Chondrosarcoma and extraskeletal chondrosarcoma are two distinct forms of sarcomas with notable differences in their origins and clinical characteristics. Chondrosarcoma typically arises from within the bones, primarily affecting the cartilage-bearing bones like the pelvis, femur, and ribs [5]. In contrast, extraskeletal chondrosarcoma develops in soft tissues such as muscles and tendons, or even in locations like the trachea or skin, making its anatomical distribution broader and less predictable [6]. Histologically, both types share similarities in their production of cartilage-like tissue, yet extraskeletal chondrosarcomas can present challenges in diagnosis due to their rarity and propensity to mimic other soft tissue sarcomas. Treatment approaches also differ: surgical excision remains the primary treatment for both, but the feasibility of complete resection and the potential need for adjuvant therapies like radiation or chemotherapy vary based on tumor location and grade [7]. In our patient, the lesion was localized within the trapezius muscle. Since the patient had no metastasis, we deemed wide resection to be the appropriate treatment.

## Conclusions

In conclusion, extraskeletal chondrosarcoma is a rare and challenging soft tissue sarcoma originating outside of bone tissue, characterized by its production of cartilage-like material. Surgical excision remains pivotal, aiming for complete tumor removal with clear margins to reduce local recurrence risk. Due to its diverse anatomical locations and potential aggressiveness, meticulous preoperative planning and multidisciplinary approaches are essential. Advances in surgical techniques, imaging, and pathology have improved treatment outcomes. Ongoing research into the genetic and molecular aspects of extraskeletal chondrosarcoma offers hope for personalized therapies, promising enhanced long-term outcomes and quality of life for patients.
